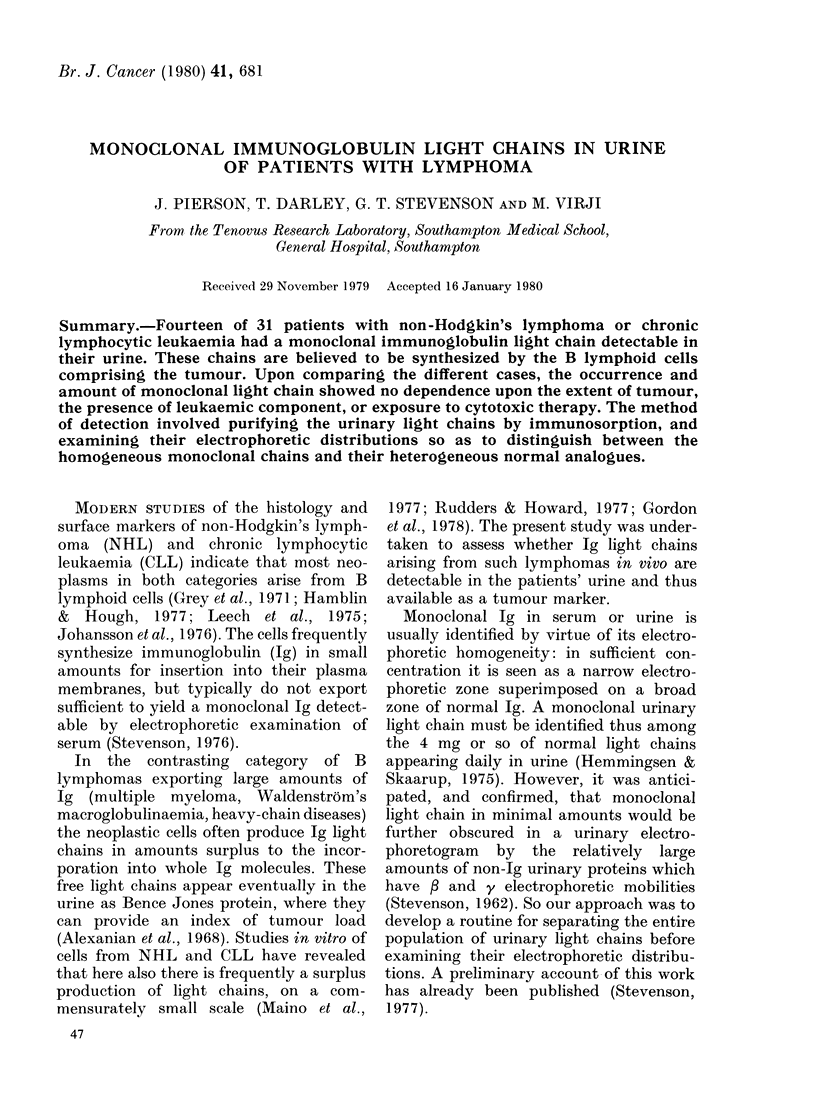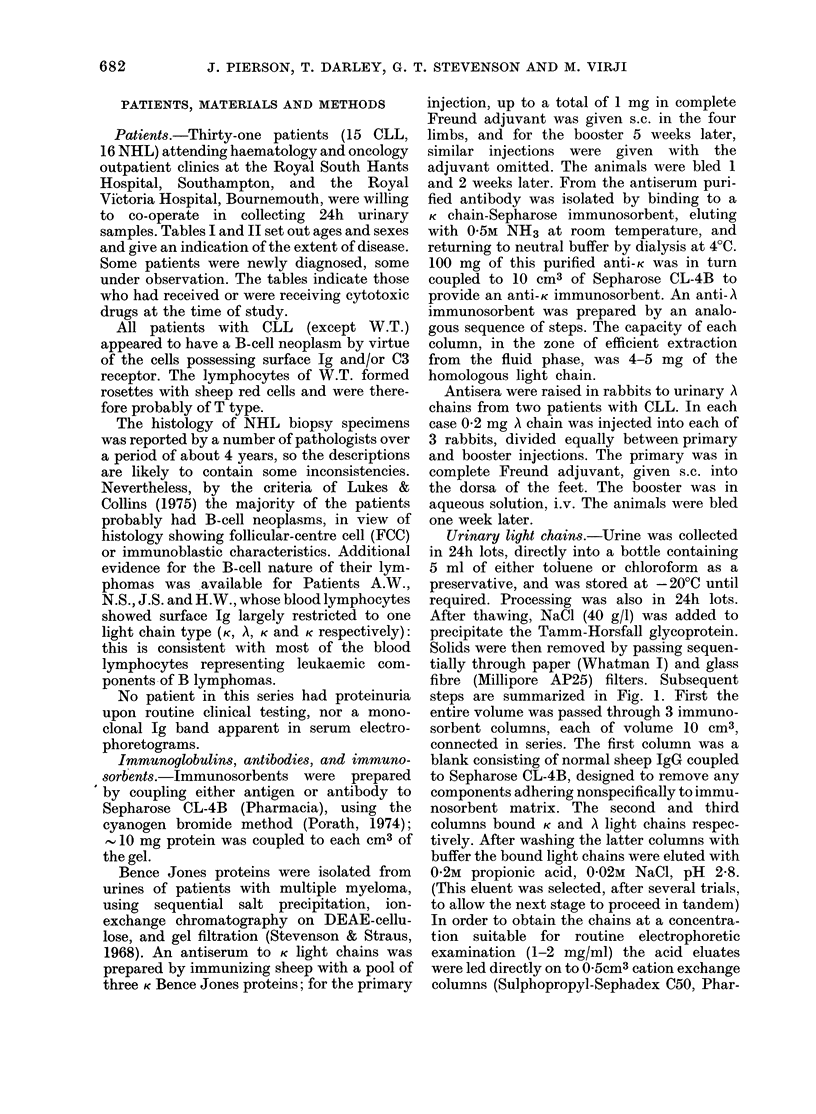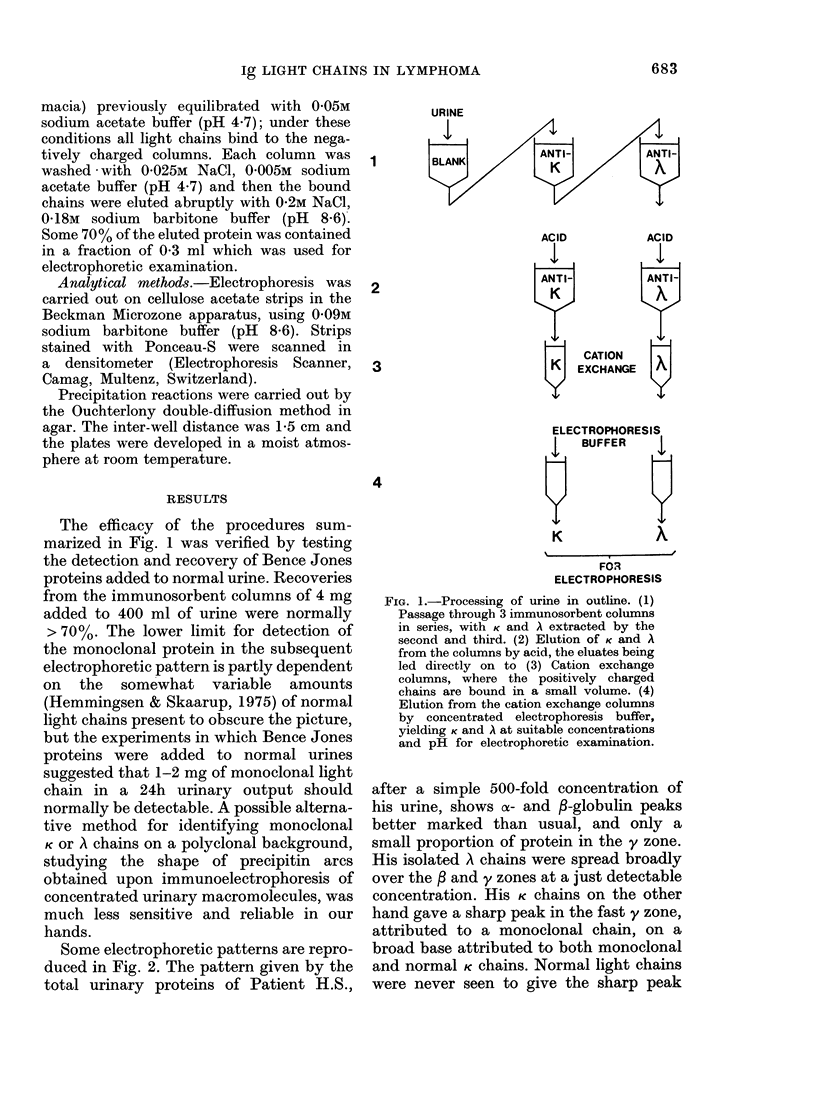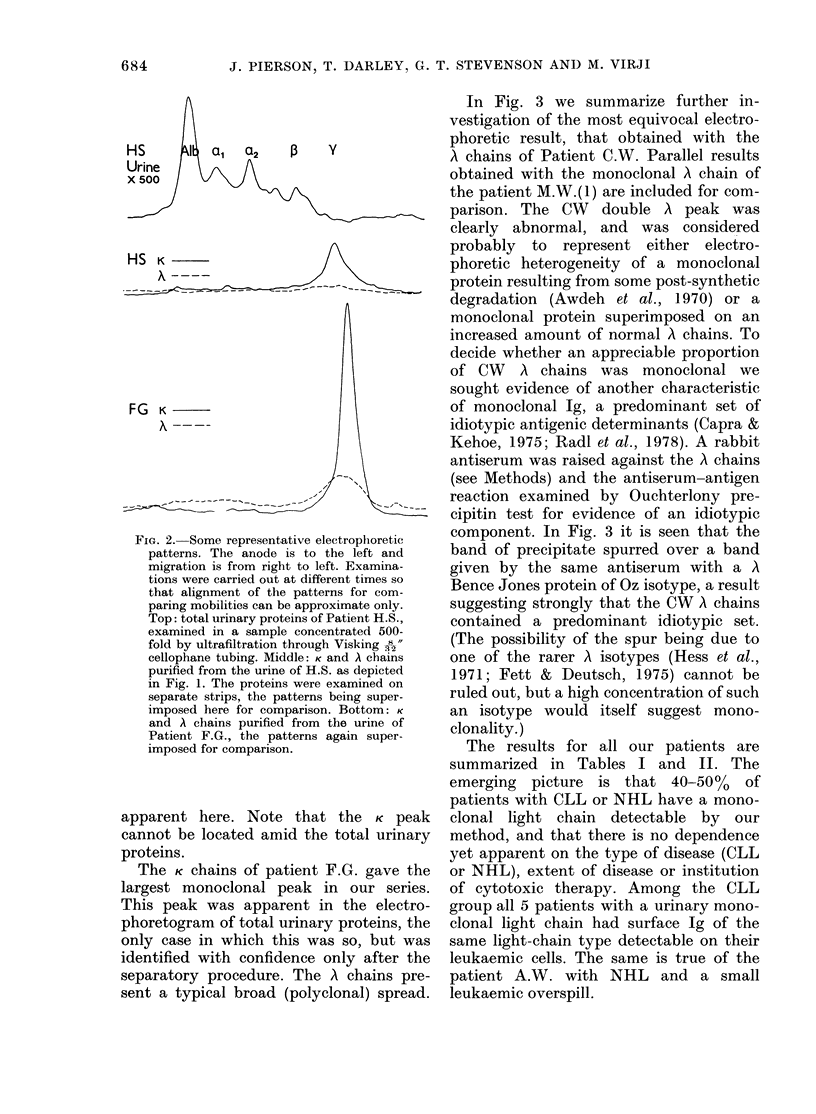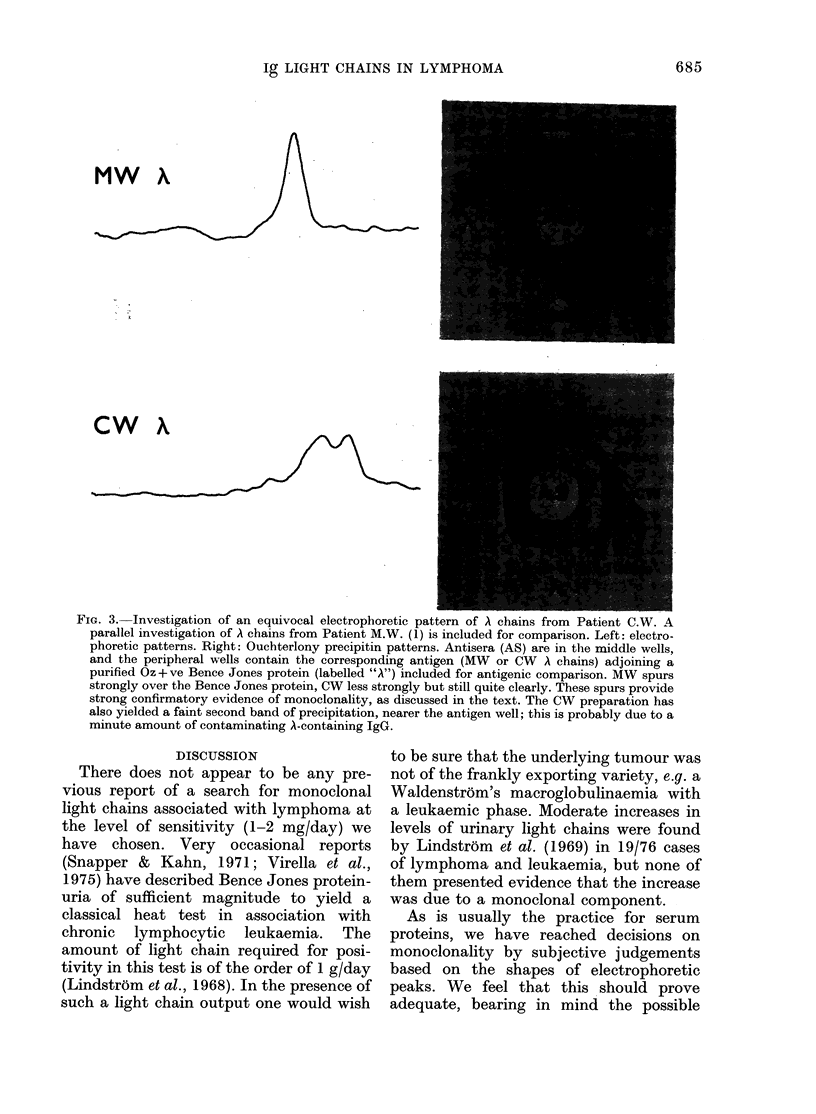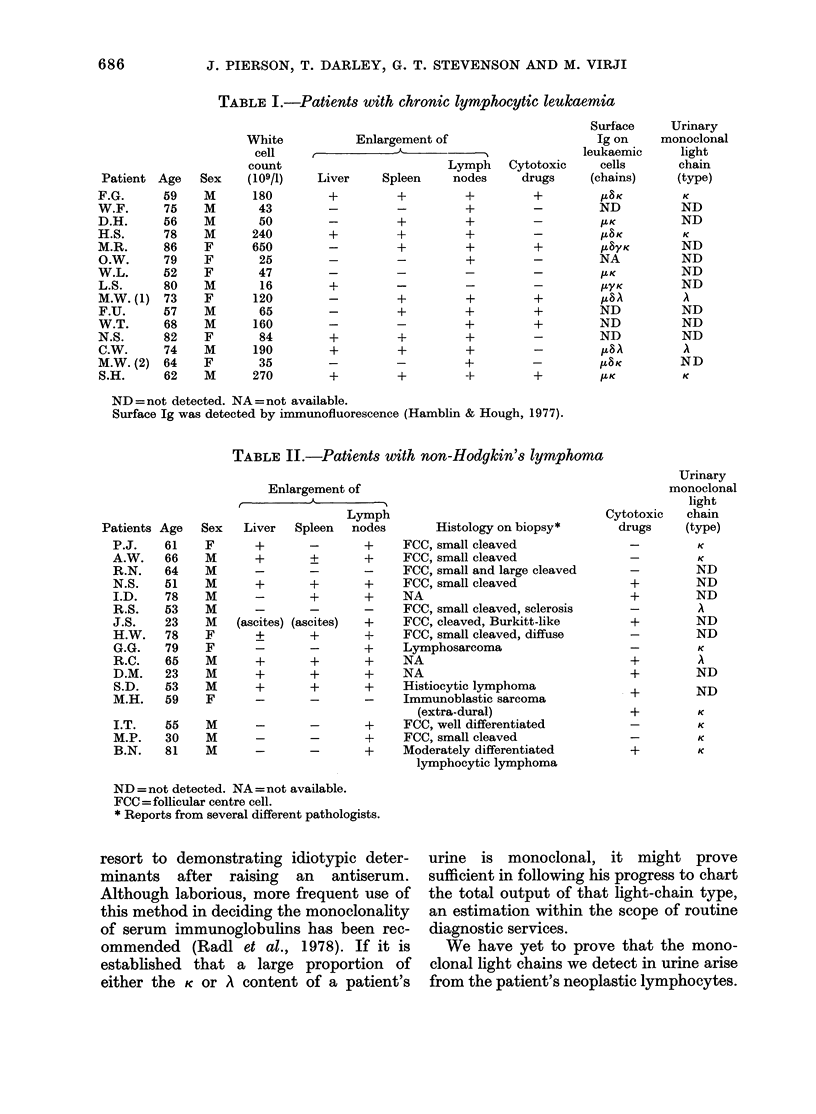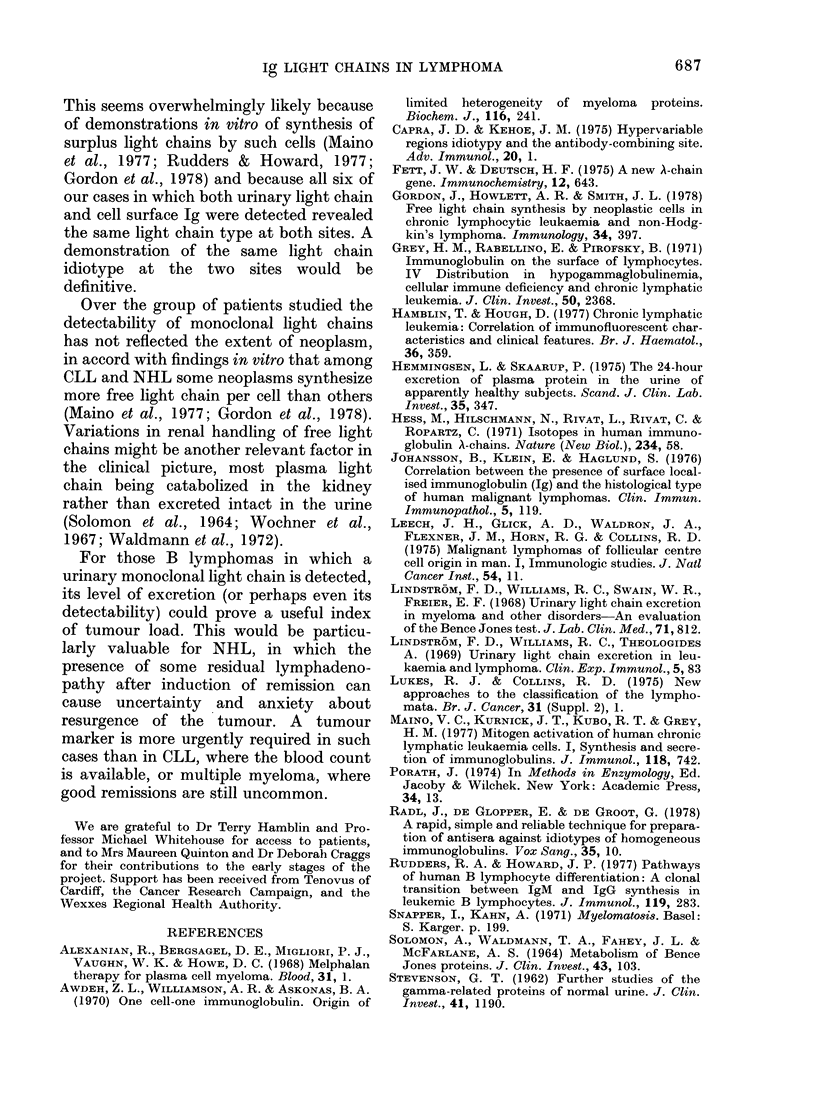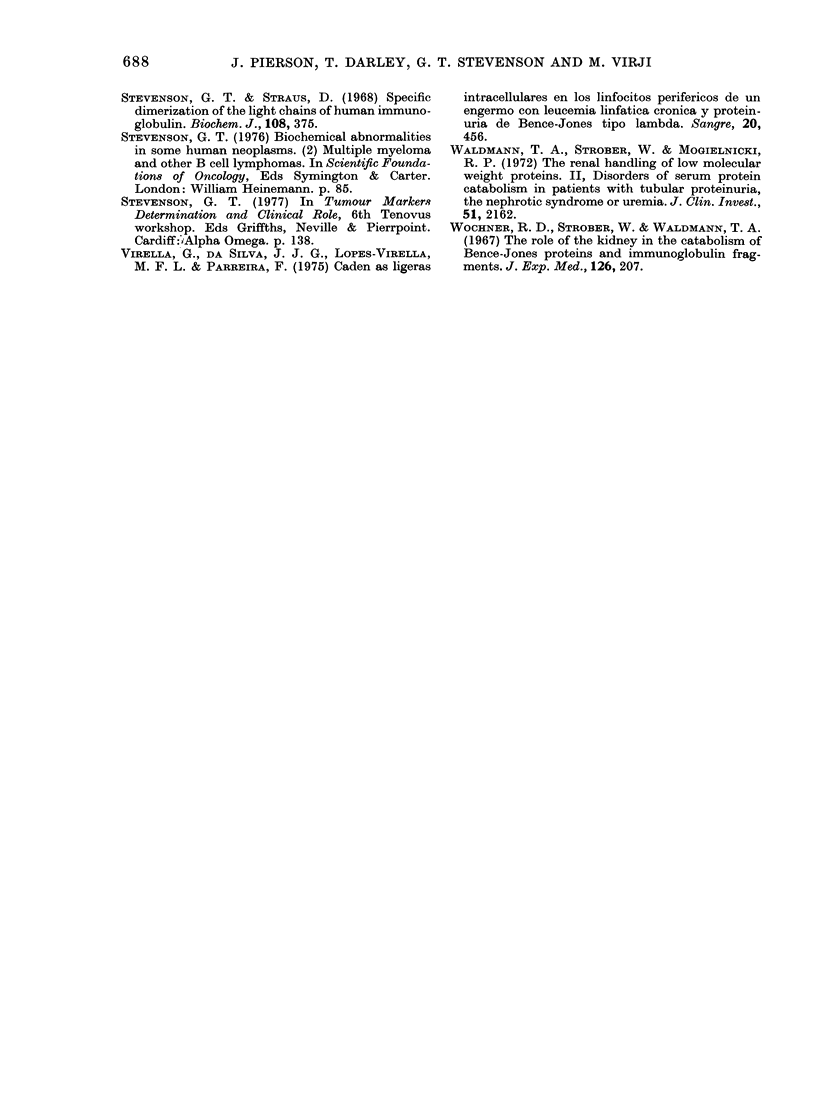# Monoclonal immunoglobulin light chains in urine of patients with lymphoma.

**DOI:** 10.1038/bjc.1980.129

**Published:** 1980-05

**Authors:** J. Pierson, T. Darley, G. T. Stevenson, M. Virji

## Abstract

**Images:**


*Br. J. Cancer* (1980) **41**, 681

### MONOCLONAL IMMUNOGLOBULIN LIGHT CHAINS IN URINE OF PATIENTS WITH LYMPHOMA

J. PIERSON, T. DARLEY, G. T. STEVENSON AND M. VIRJI

*From the Tenovus Research Laboratory, Southampton Medical School, General Hospital, Southampton*

Received 29 November 1979 Accepted 16 January 1980

**Summary.—Fourteen of 31 patients with non-Hodgkin's lymphoma or chronic lymphocytic leukaemia had a monoclonal immunoglobulin light chain detectable in their urine. These chains are believed to be synthesized by the B lymphoid cells comprising the tumour. Upon comparing the different cases, the occurrence and amount of monoclonal light chain showed no dependence upon the extent of tumour, the presence of leukaemic component, or exposure to cytotoxic therapy. The method of detection involved purifying the urinary light chains by immunosorption, and examining their electrophoretic distributions so as to distinguish between the homogeneous monoclonal chains and their heterogeneous normal analogues.**

MODERN STUDIES of the histology and surface markers of non-Hodgkin's lymphoma (NHL) and chronic lymphocytic leukaemia (CLL) indicate that most neoplasms in both categories arise from B lymphoid cells (Grey *et al.*, 1971; Hamblin & Hough, 1977; Leech *et al.*, 1975; Johansson *et al.*, 1976). The cells frequently synthesize immunoglobulin (Ig) in small amounts for insertion into their plasma membranes, but typically do not export sufficient to yield a monoclonal Ig detectable by electrophoretic examination of serum (Stevenson, 1976).

In the contrasting category of B lymphomas exporting large amounts of Ig (multiple myeloma, Waldenström's macroglobulinaemia, heavy-chain diseases) the neoplastic cells often produce Ig light chains in amounts surplus to the incorporation into whole Ig molecules. These free light chains appear eventually in the urine as Bence Jones protein, where they can provide an index of tumour load (Alexanian *et al.*, 1968). Studies *in vitro* of cells from NHL and CLL have revealed that here also there is frequently a surplus production of light chains, on a commensurately small scale (Maino *et al.*, 1977; Rudders & Howard, 1977; Gordon *et al.*, 1978). The present study was undertaken to assess whether Ig light chains arising from such lymphomas *in vivo* are detectable in the patients' urine and thus available as a tumour marker.

Monoclonal Ig in serum or urine is usually identified by virtue of its electrophoretic homogeneity: in sufficient concentration it is seen as a narrow electrophoretic zone superimposed on a broad zone of normal Ig. A monoclonal urinary light chain must be identified thus among the 4 mg or so of normal light chains appearing daily in urine (Hemmingsen & Skaarup, 1975). However, it was anticipated, and confirmed, that monoclonal light chain in minimal amounts would be further obscured in a urinary electrophoretogram by the relatively large amounts of non-Ig urinary proteins which have $\beta$ and $\gamma$ electrophoretic mobilities (Stevenson, 1962). So our approach was to develop a routine for separating the entire population of urinary light chains before examining their electrophoretic distributions. A preliminary account of this work has already been published (Stevenson, 1977).

### PATIENTS, MATERIALS AND METHODS

*Patients.*—Thirty-one patients (15 CLL, 16 NHL) attending haematology and oncology outpatient clinics at the Royal South Hants Hospital, Southampton, and the Royal Victoria Hospital, Bournemouth, were willing to co-operate in collecting 24h urinary samples. Tables I and II set out ages and sexes and give an indication of the extent of disease. Some patients were newly diagnosed, some under observation. The tables indicate those who had received or were receiving cytotoxic drugs at the time of study.

All patients with CLL (except W.T.) appeared to have a B-cell neoplasm by virtue of the cells possessing surface Ig and/or C3 receptor. The lymphocytes of W.T. formed rosettes with sheep red cells and were therefore probably of T type.

The histology of NHL biopsy specimens was reported by a number of pathologists over a period of about 4 years, so the descriptions are likely to contain some inconsistencies. Nevertheless, by the criteria of Lukes & Collins (1975) the majority of the patients probably had B-cell neoplasms, in view of histology showing follicular-centre cell (FCC) or immunoblastic characteristics. Additional evidence for the B-cell nature of their lymphomas was available for Patients A.W., N.S., J.S. and H.W., whose blood lymphocytes showed surface Ig largely restricted to one light chain type ($\kappa$, $\lambda$, $\kappa$ and $\kappa$ respectively): this is consistent with most of the blood lymphocytes representing leukaemic components of B lymphomas.

No patient in this series had proteinuria upon routine clinical testing, nor a monoclonal Ig band apparent in serum electrophoretograms.

*Immunoglobulins, antibodies, and immunosorbents.*—Immunosorbents were prepared by coupling either antigen or antibody to Sepharose CL-4B (Pharmacia), using the cyanogen bromide method (Porath, 1974); $\sim$10 mg protein was coupled to each cm$^3$ of the gel.

Bence Jones proteins were isolated from urines of patients with multiple myeloma, using sequential salt precipitation, ion-exchange chromatography on DEAE-cellulose, and gel filtration (Stevenson & Straus, 1968). An antiserum to $\kappa$ light chains was prepared by immunizing sheep with a pool of three $\kappa$ Bence Jones proteins; for the primary injection, up to a total of 1 mg in complete Freund adjuvant was given s.c. in the four limbs, and for the booster 5 weeks later, similar injections were given with the adjuvant omitted. The animals were bled 1 and 2 weeks later. From the antiserum purified antibody was isolated by binding to a $\kappa$ chain-Sepharose immunosorbent, eluting with 0·5M $NH_3$ at room temperature, and returning to neutral buffer by dialysis at 4°C. 100 mg of this purified anti-$\kappa$ was in turn coupled to 10 cm$^3$ of Sepharose CL-4B to provide an anti-$\kappa$ immunosorbent. An anti-$\lambda$ immunosorbent was prepared by an analogous sequence of steps. The capacity of each column, in the zone of efficient extraction from the fluid phase, was 4–5 mg of the homologous light chain.

Antisera were raised in rabbits to urinary $\lambda$ chains from two patients with CLL. In each case 0·2 mg $\lambda$ chain was injected into each of 3 rabbits, divided equally between primary and booster injections. The primary was in complete Freund adjuvant, given s.c. into the dorsa of the feet. The booster was in aqueous solution, i.v. The animals were bled one week later.

*Urinary light chains.*—Urine was collected in 24h lots, directly into a bottle containing 5 ml of either toluene or chloroform as a preservative, and was stored at −20°C until required. Processing was also in 24h lots. After thawing, NaCl (40 g/l) was added to precipitate the Tamm-Horsfall glycoprotein. Solids were then removed by passing sequentially through paper (Whatman I) and glass fibre (Millipore AP25) filters. Subsequent steps are summarized in Fig. 1. First the entire volume was passed through 3 immunosorbent columns, each of volume 10 cm$^3$, connected in series. The first column was a blank consisting of normal sheep IgG coupled to Sepharose CL-4B, designed to remove any components adhering nonspecifically to immunosorbent matrix. The second and third columns bound $\kappa$ and $\lambda$ light chains respectively. After washing the latter columns with buffer the bound light chains were eluted with 0·2M propionic acid, 0·02M NaCl, pH 2·8. (This eluent was selected, after several trials, to allow the next stage to proceed in tandem) In order to obtain the chains at a concentration suitable for routine electrophoretic examination (1–2 mg/ml) the acid eluates were led directly on to 0·5cm$^3$ cation exchange columns (Sulphopropyl-Sephadex C50, Pharmacia) previously equilibrated with 0·05M sodium acetate buffer (pH 4·7); under these conditions all light chains bind to the negatively charged columns. Each column was washed with 0·025M NaCl, 0·005M sodium acetate buffer (pH 4·7) and then the bound chains were eluted abruptly with 0·2M NaCl, 0·18M sodium barbitone buffer (pH 8·6). Some 70% of the eluted protein was contained in a fraction of 0·3 ml which was used for electrophoretic examination.

*Analytical methods.*—Electrophoresis was carried out on cellulose acetate strips in the Beckman Microzone apparatus, using 0·09M sodium barbitone buffer (pH 8·6). Strips stained with Ponceau-S were scanned in a densitometer (Electrophoresis Scanner, Camag, Multenz, Switzerland).

Precipitation reactions were carried out by the Ouchterlony double-diffusion method in agar. The inter-well distance was 1·5 cm and the plates were developed in a moist atmosphere at room temperature.

RESULTS

The efficacy of the procedures summarized in Fig. 1 was verified by testing the detection and recovery of Bence Jones proteins added to normal urine. Recoveries from the immunosorbent columns of 4 mg added to 400 ml of urine were normally >70%. The lower limit for detection of the monoclonal protein in the subsequent electrophoretic pattern is partly dependent on the somewhat variable amounts (Hemmingsen & Skaarup, 1975) of normal light chains present to obscure the picture, but the experiments in which Bence Jones proteins were added to normal urines suggested that 1–2 mg of monoclonal light chain in a 24h urinary output should normally be detectable. A possible alternative method for identifying monoclonal $\kappa$ or $\lambda$ chains on a polyclonal background, studying the shape of precipitin arcs obtained upon immunoelectrophoresis of concentrated urinary macromolecules, was much less sensitive and reliable in our hands.

Some electrophoretic patterns are reproduced in Fig. 2. The pattern given by the total urinary proteins of Patient H.S.,

FIG. 1.—Processing of urine in outline. (1) Passage through 3 immunosorbent columns in series, with $\kappa$ and $\lambda$ extracted by the second and third. (2) Elution of $\kappa$ and $\lambda$ from the columns by acid, the eluates being led directly on to (3) Cation exchange columns, where the positively charged chains are bound in a small volume. (4) Elution from the cation exchange columns by concentrated electrophoresis buffer, yielding $\kappa$ and $\lambda$ at suitable concentrations and pH for electrophoretic examination.

after a simple 500-fold concentration of his urine, shows $\alpha$- and $\beta$-globulin peaks better marked than usual, and only a small proportion of protein in the $\gamma$ zone. His isolated $\lambda$ chains were spread broadly over the $\beta$ and $\gamma$ zones at a just detectable concentration. His $\kappa$ chains on the other hand gave a sharp peak in the fast $\gamma$ zone, attributed to a monoclonal chain, on a broad base attributed to both monoclonal and normal $\kappa$ chains. Normal light chains were never seen to give the sharp peak

FIG. 2.—Some representative electrophoretic patterns. The anode is to the left and migration is from right to left. Examinations were carried out at different times so that alignment of the patterns for comparing mobilities can be approximate only. Top: total urinary proteins of Patient H.S., examined in a sample concentrated 500-fold by ultrafiltration through Visking $\frac{8}{32}$″ cellophane tubing. Middle: κ and λ chains purified from the urine of H.S. as depicted in Fig. 1. The proteins were examined on separate strips, the patterns being superimposed here for comparison. Bottom: κ and λ chains purified from the urine of Patient F.G., the patterns again superimposed for comparison.

apparent here. Note that the κ peak cannot be located amid the total urinary proteins.

The κ chains of patient F.G. gave the largest monoclonal peak in our series. This peak was apparent in the electrophoretogram of total urinary proteins, the only case in which this was so, but was identified with confidence only after the separatory procedure. The λ chains present a typical broad (polyclonal) spread.

In Fig. 3 we summarize further investigation of the most equivocal electrophoretic result, that obtained with the λ chains of Patient C.W. Parallel results obtained with the monoclonal λ chain of the patient M.W.(1) are included for comparison. The CW double λ peak was clearly abnormal, and was considered probably to represent either electrophoretic heterogeneity of a monoclonal protein resulting from some post-synthetic degradation (Awdeh *et al.*, 1970) or a monoclonal protein superimposed on an increased amount of normal λ chains. To decide whether an appreciable proportion of CW λ chains was monoclonal we sought evidence of another characteristic of monoclonal Ig, a predominant set of idiotypic antigenic determinants (Capra & Kehoe, 1975; Radl *et al.*, 1978). A rabbit antiserum was raised against the λ chains (see Methods) and the antiserum–antigen reaction examined by Ouchterlony precipitin test for evidence of an idiotypic component. In Fig. 3 it is seen that the band of precipitate spurred over a band given by the same antiserum with a λ Bence Jones protein of Oz isotype, a result suggesting strongly that the CW λ chains contained a predominant idiotypic set. (The possibility of the spur being due to one of the rarer λ isotypes (Hess *et al.*, 1971; Fett & Deutsch, 1975) cannot be ruled out, but a high concentration of such an isotype would itself suggest monoclonality.)

The results for all our patients are summarized in Tables I and II. The emerging picture is that 40–50% of patients with CLL or NHL have a monoclonal light chain detectable by our method, and that there is no dependence yet apparent on the type of disease (CLL or NHL), extent of disease or institution of cytotoxic therapy. Among the CLL group all 5 patients with a urinary monoclonal light chain had surface Ig of the same light-chain type detectable on their leukaemic cells. The same is true of the patient A.W. with NHL and a small leukaemic overspill.

FIG. 3.—Investigation of an equivocal electrophoretic pattern of λ chains from Patient C.W. A parallel investigation of λ chains from Patient M.W. (1) is included for comparison. Left: electrophoretic patterns. Right: Ouchterlony precipitin patterns. Antisera (AS) are in the middle wells, and the peripheral wells contain the corresponding antigen (MW or CW λ chains) adjoining a purified Oz+ve Bence Jones protein (labelled "λ") included for antigenic comparison. MW spurs strongly over the Bence Jones protein, CW less strongly but still quite clearly. These spurs provide strong confirmatory evidence of monoclonality, as discussed in the text. The CW preparation has also yielded a faint second band of precipitation, nearer the antigen well; this is probably due to a minute amount of contaminating λ-containing IgG.

### DISCUSSION

There does not appear to be any previous report of a search for monoclonal light chains associated with lymphoma at the level of sensitivity (1–2 mg/day) we have chosen. Very occasional reports (Snapper & Kahn, 1971; Virella *et al.*, 1975) have described Bence Jones proteinuria of sufficient magnitude to yield a classical heat test in association with chronic lymphocytic leukaemia. The amount of light chain required for positivity in this test is of the order of 1 g/day (Lindström *et al.*, 1968). In the presence of such a light chain output one would wish to be sure that the underlying tumour was not of the frankly exporting variety, *e.g.* a Waldenström's macroglobulinaemia with a leukaemic phase. Moderate increases in levels of urinary light chains were found by Lindström *et al.* (1969) in 19/76 cases of lymphoma and leukaemia, but none of them presented evidence that the increase was due to a monoclonal component.

As is usually the practice for serum proteins, we have reached decisions on monoclonality by subjective judgements based on the shapes of electrophoretic peaks. We feel that this should prove adequate, bearing in mind the possible

TABLE I.—*Patients with chronic lymphocytic leukaemia*

| Patient | Age | Sex | White cell count ($10^9$/l) | Enlargement of: Liver | Spleen | Lymph nodes | Cytotoxic drugs | Surface Ig on leukaemic cells (chains) | Urinary monoclonal light chain (type) |
|---|---|---|---|---|---|---|---|---|---|
| F.G. | 59 | M | 180 | + | + | + | + | μδκ | κ |
| W.F. | 75 | M | 43 | − | − | + | − | ND | ND |
| D.H. | 56 | M | 50 | − | + | + | − | μκ | ND |
| H.S. | 78 | M | 240 | + | + | + | − | μδκ | κ |
| M.R. | 86 | F | 650 | − | + | + | + | μδγκ | ND |
| O.W. | 79 | F | 25 | − | − | + | − | NA | ND |
| W.L. | 52 | F | 47 | − | − | − | − | μκ | ND |
| L.S. | 80 | M | 16 | + | − | − | − | μγκ | ND |
| M.W. (1) | 73 | F | 120 | − | + | + | + | μδλ | λ |
| F.U. | 57 | M | 65 | − | + | + | + | ND | ND |
| W.T. | 68 | M | 160 | − | − | + | + | ND | ND |
| N.S. | 82 | F | 84 | + | + | + | − | ND | ND |
| C.W. | 74 | M | 190 | + | + | + | − | μδλ | λ |
| M.W. (2) | 64 | F | 35 | − | − | + | − | μδκ | ND |
| S.H. | 62 | M | 270 | + | + | + | + | μκ | κ |

ND = not detected. NA = not available.
Surface Ig was detected by immunofluorescence (Hamblin & Hough, 1977).

TABLE II.—*Patients with non-Hodgkin's lymphoma*

| Patients | Age | Sex | Enlargement of: Liver | Spleen | Lymph nodes | Histology on biopsy* | Cytotoxic drugs | Urinary monoclonal light chain (type) |
|---|---|---|---|---|---|---|---|---|
| P.J. | 61 | F | + | − | + | FCC, small cleaved | − | κ |
| A.W. | 66 | M | + | ± | + | FCC, small cleaved | − | κ |
| R.N. | 64 | M | − | − | − | FCC, small and large cleaved | − | ND |
| N.S. | 51 | M | + | + | + | FCC, small cleaved | + | ND |
| I.D. | 78 | M | − | + | + | NA | + | ND |
| R.S. | 53 | M | − | − | − | FCC, small cleaved, sclerosis | − | λ |
| J.S. | 23 | M | (ascites) | (ascites) | + | FCC, cleaved, Burkitt-like | + | ND |
| H.W. | 78 | F | ± | + | + | FCC, small cleaved, diffuse | − | ND |
| G.G. | 79 | F | − | − | + | Lymphosarcoma | − | κ |
| R.C. | 65 | M | + | + | + | NA | + | λ |
| D.M. | 23 | M | + | + | + | NA | + | ND |
| S.D. | 53 | M | + | + | + | Histiocytic lymphoma | + | ND |
| M.H. | 59 | F | − | − | − | Immunoblastic sarcoma | | |
| | | | | | | (extra-dural) | + | κ |
| I.T. | 55 | M | − | − | + | FCC, well differentiated | − | κ |
| M.P. | 30 | M | − | − | + | FCC, small cleaved | − | κ |
| B.N. | 81 | M | − | − | + | Moderately differentiated lymphocytic lymphoma | + | κ |

ND = not detected. NA = not available.
FCC = follicular centre cell.
* Reports from several different pathologists.

resort to demonstrating idiotypic determinants after raising an antiserum. Although laborious, more frequent use of this method in deciding the monoclonality of serum immunoglobulins has been recommended (Radl *et al.*, 1978). If it is established that a large proportion of either the κ or λ content of a patient's urine is monoclonal, it might prove sufficient in following his progress to chart the total output of that light-chain type, an estimation within the scope of routine diagnostic services.

We have yet to prove that the monoclonal light chains we detect in urine arise from the patient's neoplastic lymphocytes.

This seems overwhelmingly likely because of demonstrations *in vitro* of synthesis of surplus light chains by such cells (Maino *et al.*, 1977; Rudders & Howard, 1977; Gordon *et al.*, 1978) and because all six of our cases in which both urinary light chain and cell surface Ig were detected revealed the same light chain type at both sites. A demonstration of the same light chain idiotype at the two sites would be definitive.

Over the group of patients studied the detectability of monoclonal light chains has not reflected the extent of neoplasm, in accord with findings *in vitro* that among CLL and NHL some neoplasms synthesize more free light chain per cell than others (Maino *et al.*, 1977; Gordon *et al.*, 1978). Variations in renal handling of free light chains might be another relevant factor in the clinical picture, most plasma light chain being catabolized in the kidney rather than excreted intact in the urine (Solomon *et al.*, 1964; Wochner *et al.*, 1967; Waldmann *et al.*, 1972).

For those B lymphomas in which a urinary monoclonal light chain is detected, its level of excretion (or perhaps even its detectability) could prove a useful index of tumour load. This would be particularly valuable for NHL, in which the presence of some residual lymphadenopathy after induction of remission can cause uncertainty and anxiety about resurgence of the tumour. A tumour marker is more urgently required in such cases than in CLL, where the blood count is available, or multiple myeloma, where good remissions are still uncommon.

We are grateful to Dr Terry Hamblin and Professor Michael Whitehouse for access to patients, and to Mrs Maureen Quinton and Dr Deborah Craggs for their contributions to the early stages of the project. Support has been received from Tenovus of Cardiff, the Cancer Research Campaign, and the Wexxes Regional Health Authority.

### REFERENCES

ALEXANIAN, R., BERGSAGEL, D. E., MIGLIORI, P. J., VAUGHN, W. K. & HOWE, D. C. (1968) Melphalan therapy for plasma cell myeloma. *Blood*, **31**, 1.

AWDEH, Z. L., WILLIAMSON, A. R. & ASKONAS, B. A. (1970) One cell-one immunoglobulin. Origin of limited heterogeneity of myeloma proteins. *Biochem. J.*, **116**, 241.

CAPRA, J. D. & KEHOE, J. M. (1975) Hypervariable regions idiotypy and the antibody-combining site. *Adv. Immunol.*, **20**, 1.

FETT, J. W. & DEUTSCH, H. F. (1975) A new λ-chain gene. *Immunochemistry*, **12**, 643.

GORDON, J., HOWLETT, A. R. & SMITH, J. L. (1978) Free light chain synthesis by neoplastic cells in chronic lymphocytic leukaemia and non-Hodgkin's lymphoma. *Immunology*, **34**, 397.

GREY, H. M., RABELLINO, E. & PIROFSKY, B. (1971) Immunoglobulin on the surface of lymphocytes. IV Distribution in hypogammaglobulinemia, cellular immune deficiency and chronic lymphatic leukemia. *J. Clin. Invest.*, **50**, 2368.

HAMBLIN, T. & HOUGH, D. (1977) Chronic lymphatic leukemia: Correlation of immunofluorescent characteristics and clinical features. *Br. J. Haematol.*, **36**, 359.

HEMMINGSEN, L. & SKAARUP, P. (1975) The 24-hour excretion of plasma protein in the urine of apparently healthy subjects. *Scand. J. Clin. Lab. Invest.*, **35**, 347.

HESS, M., HILSCHMANN, N., RIVAT, L., RIVAT, C. & ROPARTZ, C. (1971) Isotopes in human immunoglobulin λ-chains. *Nature (New Biol.)*, **234**, 58.

JOHANSSON, B., KLEIN, E. & HAGLUND, S. (1976) Correlation between the presence of surface localised immunoglobulin (Ig) and the histological type of human malignant lymphomas. *Clin. Immun. Immunopathol.*, **5**, 119.

LEECH, J. H., GLICK, A. D., WALDRON, J. A., FLEXNER, J. M., HORN, R. G. & COLLINS, R. D. (1975) Malignant lymphomas of follicular centre cell origin in man. I, Immunologic studies. *J. Natl Cancer Inst.*, **54**, 11.

LINDSTRÖM, F. D., WILLIAMS, R. C., SWAIN, W. R., FREIER, E. F. (1968) Urinary light chain excretion in myeloma and other disorders—An evaluation of the Bence Jones test. *J. Lab. Clin. Med.*, **71**, 812.

LINDSTRÖM, F. D., WILLIAMS, R. C., THEOLOGIDES A. (1969) Urinary light chain excretion in leukaemia and lymphoma. *Clin. Exp. Immunol.*, **5**, 83

LUKES, R. J. & COLLINS, R. D. (1975) New approaches to the classification of the lymphomata. *Br. J. Cancer*, **31** (Suppl. 2), 1.

MAINO, V. C., KURNICK, J. T., KUBO, R. T. & GREY, H. M. (1977) Mitogen activation of human chronic lymphatic leukaemia cells. I, Synthesis and secretion of immunoglobulins. *J. Immunol.*, **118**, 742.

PORATH, J. (1974) In *Methods in Enzymology*, Ed. Jacoby & Wilchek. New York: Academic Press, **34**, 13.

RADL, J., DE GLOPPER, E. & DE GROOT, G. (1978) A rapid, simple and reliable technique for preparation of antisera against idiotypes of homogeneous immunoglobulins. *Vox Sang.*, **35**, 10.

RUDDERS, R. A. & HOWARD, J. P. (1977) Pathways of human B lymphocyte differentiation: A clonal transition between IgM and IgG synthesis in leukemic B lymphocytes. *J. Immunol.*, **119**, 283.

SNAPPER, I., KAHN, A. (1971) *Myelomatosis*. Basel: S. Karger. p. 199.

SOLOMON, A., WALDMANN, T. A., FAHEY, J. L. & MCFARLANE, A. S. (1964) Metabolism of Bence Jones proteins. *J. Clin. Invest.*, **43**, 103.

STEVENSON, G. T. (1962) Further studies of the gamma-related proteins of normal urine. *J. Clin. Invest.*, **41**, 1190.

STEVENSON, G. T. & STRAUS, D. (1968) Specific dimerization of the light chains of human immunoglobulin. *Biochem. J.*, **108**, 375.

STEVENSON, G. T. (1976) Biochemical abnormalities in some human neoplasms. (2) Multiple myeloma and other B cell lymphomas. In *Scientific Foundations of Oncology*, Eds Symington & Carter. London: William Heinemann. p. 85.

STEVENSON, G. T. (1977) In *Tumour Markers Determination and Clinical Role*, 6th Tenovus workshop. Eds Griffths, Neville & Pierrpoint. Cardiff: Alpha Omega. p. 138.

VIRELLA, G., DA SILVA, J. J. G., LOPES-VIRELLA, M. F. L. & PARREIRA, F. (1975) Caden as ligeras intracellulares en los linfocitos perifericos de un engermo con leucemia linfatica cronica y proteinuria de Bence-Jones tipo lambda. *Sangre*, **20**, 456.

WALDMANN, T. A., STROBER, W. & MOGIELNICKI, R. P. (1972) The renal handling of low molecular weight proteins. II, Disorders of serum protein catabolism in patients with tubular proteinuria, the nephrotic syndrome or uremia. *J. Clin. Invest.*, **51**, 2162.

WOCHNER, R. D., STROBER, W. & WALDMANN, T. A. (1967) The role of the kidney in the catabolism of Bence-Jones proteins and immunoglobulin fragments. *J. Exp. Med.*, **126**, 207.